# Advanced Photodegradation of Azo Dye Methyl Orange Using H_2_O_2_-Activated Fe_3_O_4_@SiO_2_@ZnO Composite under UV Treatment

**DOI:** 10.3390/molecules29061190

**Published:** 2024-03-07

**Authors:** Oksana Makota, Erika Dutková, Jaroslav Briančin, Jozef Bednarcik, Maksym Lisnichuk, Iryna Yevchuk, Inna Melnyk

**Affiliations:** 1 Institute of Geotechnics, Slovak Academy of Sciences, Watsonova 45, 04001 Kosice, Slovakia; dutkova@saske.sk (E.D.); briancin@saske.sk (J.B.); 2 Institute of Chemistry and Chemical Technologies, Lviv Polytechnic National University, Stepana Bandery 12, 79013 Lviv, Ukraine; 3 Faculty of Science, Pavol Jozef Šafárik University in Kosice, Park Angelinum 9, 04001 Kosice, Slovakia; jozef.bednarcik@upjs.sk (J.B.); maksym.lisnichuk@upjs.sk (M.L.); 4 Institute of Materials Research, Slovak Academy of Sciences, Watsonova 47, 04001 Kosice, Slovakia; 5 Department of Physical Chemistry of Fossil Fuels, Institute of Physical-Organic Chemistry and Coal Chemistry named after L. M. Lytvynenko, National Academy of Sciences of Ukraine, Naukova 3a, 79060 Lviv, Ukraine; irynayevchuk@gmail.com

**Keywords:** photocatalytic degradation, methyl orange, magnetic nanocomposite, H_2_O_2_ activation

## Abstract

The Fe_3_O_4_@SiO_2_@ZnO composite was synthesized via the simultaneous deposition of SiO_2_ and ZnO onto pre-prepared Fe_3_O_4_ nanoparticles. Physicochemical methods (TEM, EDXS, XRD, SEM, FTIR, PL, zeta potential measurements, and low-temperature nitrogen adsorption/desorption) revealed that the simultaneous deposition onto magnetite surfaces, up to 18 nm in size, results in the formation of an amorphous shell composed of a mixture of zinc and silicon oxides. This composite underwent modification to form Fe_3_O_4_@SiO_2_@ZnO*, achieved by activation with H_2_O_2_. The modified composite retained its structural integrity, but its surface groups underwent significant changes, exhibiting pronounced catalytic activity in the photodegradation of methyl orange under UV irradiation. It was capable of degrading 96% of this azo dye in 240 min, compared to the initial Fe_3_O_4_@SiO_2_@ZnO composite, which could remove only 11% under identical conditions. Fe_3_O_4_@SiO_2_@ZnO* demonstrated robust stability after three cycles of use in dye photodegradation. Furthermore, Fe_3_O_4_@SiO_2_@ZnO* exhibited decreased PL intensity, indicating an enhanced efficiency in electron-hole pair separation and a reduced recombination rate in the modified composite. The activation process diminishes the electron-hole (e^−^)/(h^+^) recombination and generates the potent oxidizing species, hydroxyl radicals (OH˙), on the photocatalyst surface, thereby playing a crucial role in the enhanced photodegradation efficiency of methyl orange with Fe_3_O_4_@SiO_2_@ZnO*.

## 1. Introduction

Water is essential for all life forms and serves as the foundation of civilization on our planet. It plays a critical role in nearly every life process on Earth and fulfills various functions within the climate system. Living organisms primarily consist of water, which performs numerous functions, challenging the notion of it being merely an inactive diluent [1,2,3]. When water is of inadequate quality, it results in the gradual demise of plants and animals. In recent years, certain organic chemicals, such as dyes, have become major pollutants. They are extensively used in textiles, plastics, leather, paper, pharmaceuticals, food, and cosmetics industries [4,5,6,7,8]. Their improper disposal leads to the discharge of these chemicals into groundwater and surface environments. Dye wastewater, particularly due to its high toxicity and chemical oxygen demand, causes serious environmental pollution [5,6,7,8,9,10]. Methyl orange (MO), a member of the azo dye category, is one of the most widely used organic dyes. Its complex aromatic structure makes it difficult to degrade [11,12,13,14,15,16,17,18].

Therefore, purifying water from such hazardous substances has become a critical humanitarian objective and a key area of research. Among the different wastewater treatment processes, photocatalysis attracts significant attention as a promising technology for removing dye contaminants [14,15,16,19,20]. Photocatalysis is a ‘green chemistry’ technology that employs semiconductors as photocatalysts to catalyze the degradation of toxic pollutants using a light source [11,21,22,23,24,25]. The advantages of this method include its low cost, simple construction, ease of handling, high capability, and eco-friendliness.

Photocatalysts play a key role in pollutant removal. Among the various semiconductor photocatalysts, zinc oxide (ZnO) is most frequently applied due to its photocatalytic activity, photosensitivity, low cost, non-toxic nature, and stability [10,11,12,16,17,18,23,26]. ZnO exhibits a special electronic band structure; a direct and broad band gap of 3.37 eV with an excitation binding energy of 60 meV [26,27]. However, the practical application of a single ZnO photocatalyst is still somewhat limited due to the challenges in separating and recycling its powder from treated solutions, as its small size results in wastage, higher wastewater treatment costs, and potential secondary pollution. This issue can be addressed by immobilizing ZnO on a supporting material, such as magnetite Fe_3_O_4_, which has a high magnetic function and can be easily separated and removed from water solutions by an external magnetic field [28,29,30,31,32,33,34,35,36]. However, Fe_3_O_4_ is unstable in acidic conditions and tends to agglomerate. To prevent this, it is necessary to coat the magnetite with inert silica (SiO_2_) [29,30,31,32,33,34,35,36]. Consequently, numerous magnetic photocatalysts based on ZnO have been developed by combining it with Fe_3_O_4_ or both Fe_3_O_4_ and SiO_2_ [28,29,30,31,32,33,34,35,36].

However, the main disadvantage limiting the effective performance of photocatalysts in dye photodegradation is the recombination of photogenerated electron-hole pairs [12,16,18,19,34,36,37,38]. A possible way to reduce this recombination process is by combining photocatalysts with H_2_O_2_, which acts as an electron acceptor and can capture electrons [39,40]. Moreover, activating photocatalysts with H_2_O_2_ can offer advantages such as prolonging the lifespan of the formed charge carriers and facilitating the generation of hydroxyl radicals. These radicals are highly active potential oxidizing agents, playing an important role in the degradation of contaminants [39,41,42]. In addition, H_2_O_2_ can be considered an economically viable and relatively environmentally safe compound [39]. Therefore, modifying photocatalysts with H_2_O_2_ is a promising and prospective method to improve photocatalytic efficiency in the degradation of dyes. The novelty of this research is to make magnetic (magnetite) photocatalysts (oxide shells of silica and zinc oxide) for repeated use in the photodegradation of azo dyes. The effective activation of the photocatalyst can be carried out using very low concentrations of hydrogen peroxide.

In this study, Fe_3_O_4_@SiO_2_@ZnO was synthesized through a two-step, facile method involving the simultaneous deposition of SiO_2_ and ZnO onto magnetite Fe_3_O_4_. Furthermore, a simple modification method was developed to increase its photocatalytic activity, which required only a tiny amount of H_2_O_2_. The composites, before and after modification, were studied for their morphology and crystal structure. The applied modification method led to a decrease in photoluminescence (PL) intensity due to a higher separation and lower recombination of charge carriers. Methyl orange, used as a model azo dye, underwent photodegradation under UV treatment to test the performance of the prepared composites and compare their photodegradation efficiency before and after modification. The modified composite exhibited significantly higher photocatalytic efficiency for MO. Fe_3_O_4_@SiO_2_@ZnO* showed good photocatalytic activity after three cycles of dye removal. The investigation into the effect of scavengers revealed that the created holes and hydroxyl radicals were the main reactive species involved in the photodegradation process of MO by Fe_3_O_4_@SiO_2_@ZnO*. The possible mechanism of photocatalytic degradation of MO in the presence of modified Fe_3_O_4_@SiO_2_@ZnO* is proposed and discussed in this manuscript.

## 2. Results and Discussion

### 2.1. Morphology

**Figure 1:**
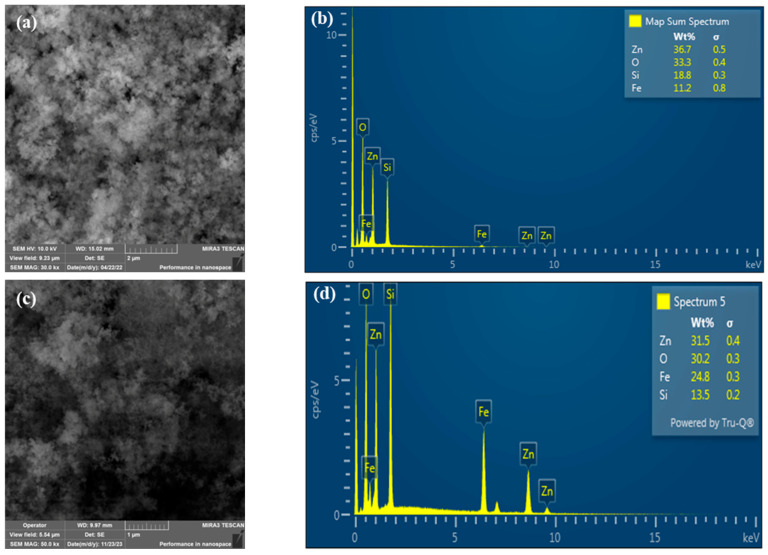
SEM images of Fe_3_O_4_@SiO_2_@ZnO (**a**) and Fe_3_O_4_@SiO_2_@ZnO* (**c**), and EDX spectra of Fe_3_O_4_@SiO_2_@ZnO (**b**) and Fe_3_O_4_@SiO_2_@ZnO* (**d**).

The morphological structure of the synthesized Fe_3_O_4_@SiO_2_@ZnO and Fe_3_O_4_@SiO_2_@ZnO* composites was observed using scanning and electron microscopy (SEM). The results of the SEM and Energy-dispersive X-ray spectroscopy (EDXS) analyses of the composites are presented in Figure 1. It can be seen that both composites display spherical particles. The size of these particles is difficult to determine, but they form agglomerates with a diameter of 400–500 nm (Figure 1a). According to [31], the SiO_2_ coating on the magnetite Fe_3_O_4_ surface could lead to the formation of a granular surface, whereas the ZnO coating might result in the agglomeration of composites and cause deformations in the particles. The EDXS analysis (Figure 1b) confirmed the presence of iron, silicon, zinc, and oxygen in the synthesized Fe_3_O_4_@SiO_2_@ZnO composite. According to the EDXS data, the distribution of elements changed slightly, indicating a higher amount of iron after activation (Figure 1d). Additionally, the reaction with H_2_O_2_ can cause physical changes in the composite, such as delamination, cracks, or pores, which can alter the way elements are distributed or appear on the surface.

**Figure 2:**
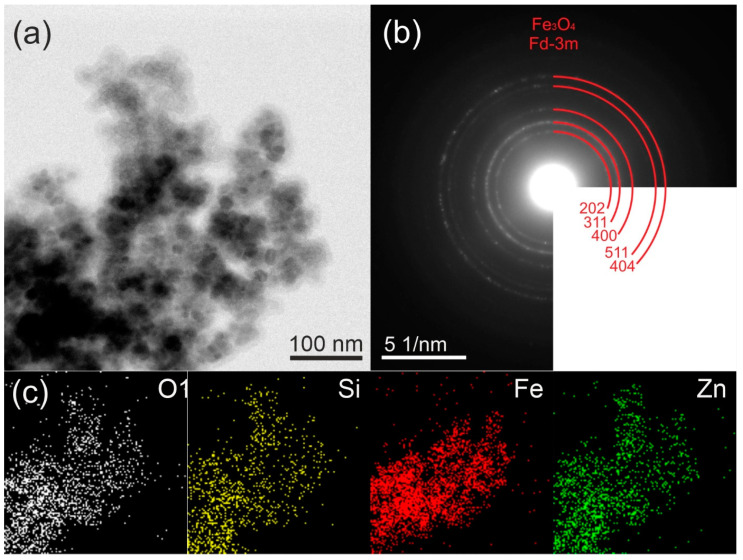
TEM images (**a**), electron diffraction patterns (**b**), and corresponding EDX analysis (**c**) of Fe_3_O_4_@SiO_2_@ZnO composite.

**Figure 3:**
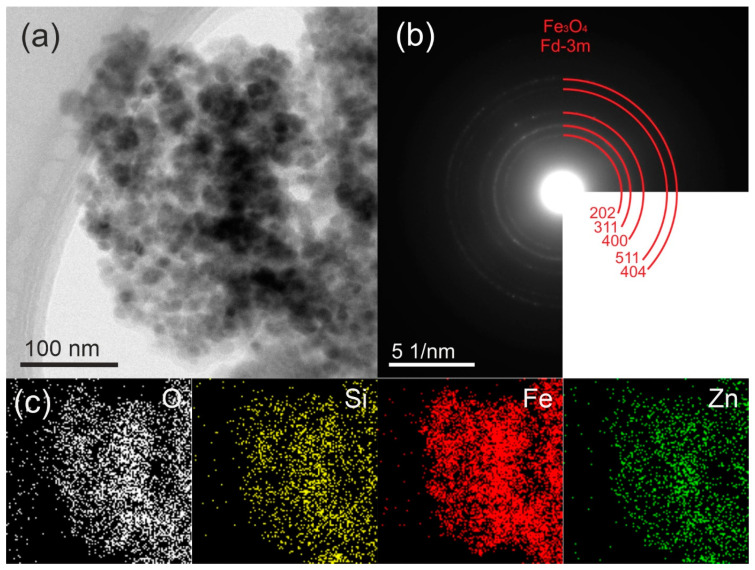
TEM images (**a**), electron diffraction patterns (**b**), and corresponding EDX analysis (**c**) of Fe_3_O_4_@SiO_2_@ZnO* composite.

Figure 2a and Figure 3a reveal the TEM micrograph of the synthesized composite materials. The dark black color represents the Fe_3_O_4_ nanoparticles (15–18 nm) while the light-dark color corresponds to the polymeric SiO_2_@ZnO particles. Here, the Fe_3_O_4_ nanoparticles acted as the core, and the polymeric SiO_2_@ZnO acted as the shell. The Fe_3_O_4_ nanoparticles were coated by the SiO_2_@ZnO nanoparticles and led to the successful formation of Fe_3_O_4_@SiO_2_@ZnO composite.

The selected-area electron diffraction (SAED) pattern, which is shown in Figure 2b, confirmed the polycrystalline character of the sample and corresponds with the results of the XRD. The SAED analysis revealed distinctive peaks associated with the crystalline planes (202), (311), (400), (511), and (404) of magnetite Fe_3_O_4_. Notably, no discernible peaks corresponding to the hexagonal structure of ZnO were observed. Moreover, the SAED pattern exhibited a diffuse halo, suggesting the amorphous nature of ZnO and SiO_2_. The EDX maps in Figure 2c showed that the distributions of Fe, Si, Zn, and O were uniform.

In the TEM micrographs and SAED patterns depicted in Figure 3b, the modified composite exhibited the same characteristics as the original composite. This suggests the preservation of the Fe_3_O_4_@SiO_2_@ZnO* phase after activation, with no noticeable changes in morphology.

### 2.2. FT-IR Spectroscopy

**Figure 4:**
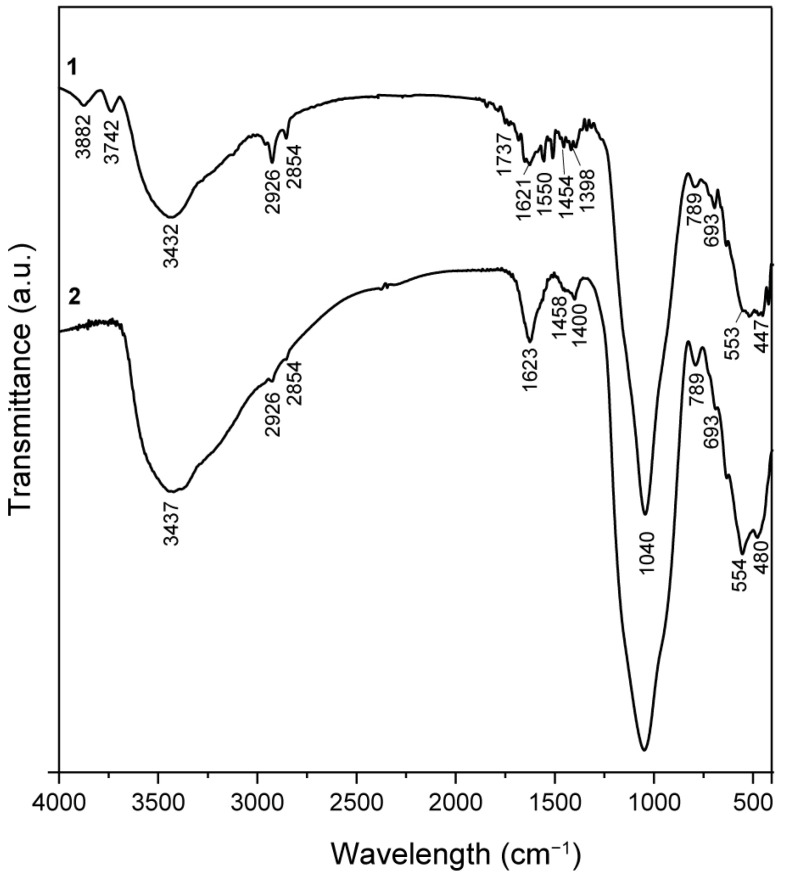
FTIR spectra of the synthesized Fe_3_O_4_@SiO_2_@ZnO (1) and Fe_3_O_4_@SiO_2_@ZnO* (2) composites.

The infrared spectra of the synthesized Fe_3_O_4_@SiO_2_@ZnO and Fe_3_O_4_@SiO_2_@ZnO* composites are shown in Figure 4. Firstly, the initial sample (spectrum 1) contained bands at 3882 cm^−1^ and 3742 cm^−1^, attributed to the stretching vibrations of O–H. There were also bands at 693 cm^−1^, relevant to -OH bending vibrations, which indicated the presence of free -OH groups on the surface of oxides. Concurrently, following the activation of the sample in an aqueous environment, these bands became indistinct or disappeared entirely. This effect was attributed to the substantial presence of water on the surface, as evidenced in spectrum 2. The wide absorption band centered at 3432 cm^−1^ corresponded to the O–H stretching modes of surface-adsorbed molecular water [34] at both spectra. The peaks in the region of 2973–2854 cm^−1^ were related to the symmetric and asymmetric stretching vibrations of –CH_3_ and –CH_2_ groups [31,43] from non-hydrolyzed residues of the starting substances. The low-intensity bands located at 1737 cm^−1^ and ~1400 cm^−1^ were assigned to the stretching vibrations of C=O and the bending mode of C–O–H, respectively [31,44], from carboxyl groups on the ZnO surface.

The absorption bands at 1550 and 1503 cm^−1^ referred to the deformation vibrations of CH_2_ and CH_3_, and, at 789 cm^−1^, referred to the CH_2_ rocking vibrations, which, like the stretching ones, belonged to the organic groups of underhydrolyzed reagents. The characteristic absorption peak at 1040 cm^−1^ was assigned to the Si–O–Si stretching vibrations [45,46]. The characteristic broad band from peaks around 447 cm^−1^ and 553 cm^−1^ were attributed to the stretching vibrations of Zn–O and Fe–O, representing the characteristic phases of ZnO and Fe_3_O_4_, respectively [46,47,48]. After activation, the absorption band corresponding to Zn–O shifted to 480 cm^−1^. Therefore, activation can induce structural changes, such as the formation of vacancies or atomic displacements, which may alter the Zn-O bond vibrations. Such changes in the lattice can affect the bonds between zinc and oxygen atoms, shifting the vibrational frequency higher in the spectrum.

### 2.3. The Phase Analysis Using XRD

**Figure 5:**
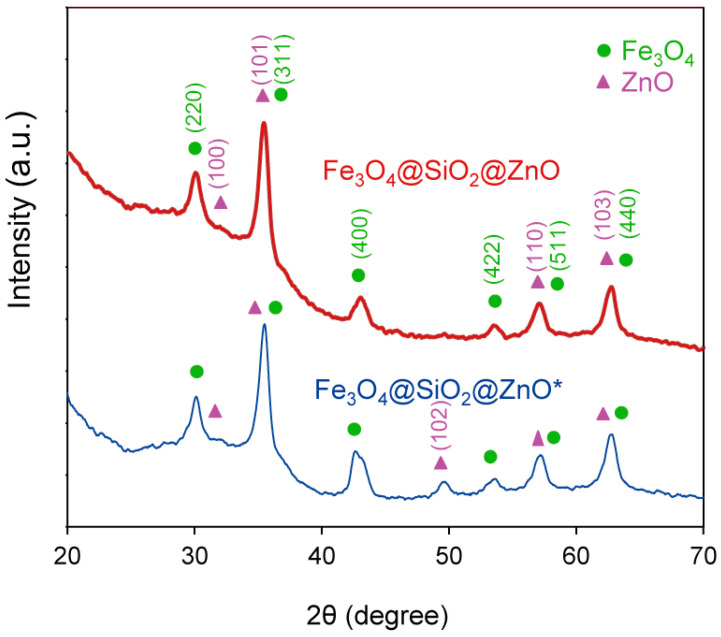
X-ray diffraction patterns of the Fe_3_O_4_@SiO_2_@ZnO and Fe_3_O_4_@SiO_2_@ZnO* composites.

The composition and crystallinity of the synthesized composite Fe_3_O_4_@SiO_2_@ZnO and its modified form Fe_3_O_4_@SiO_2_@ZnO* were investigated using XRD analysis. The obtained XRD patterns are shown in Figure 5. In the case of both composites, six peaks were observed at 2θ = 29.97°, 35.31°, 42.39°, 52.82°, 56.71°, and 62.41°. These were assigned to the (220), (311), (400), (422), (511), and (440) crystal planes of magnetite Fe_3_O_4_ (JCPDS card No. 19-0629). This was consistent with the TEM data (Figure 2 and Figure 3). Additionally, low-intensity peaks at 31.16°, 35.31°, 49.12°, 56.70°, and 62.42° were indexed to the (100), (101), (102), (110), and (103) crystal planes, corresponding to the hexagonal wurtzite structure of ZnO (JCPDS card No. 36-1451). SiO_2_ is amorphous; thus, no diffraction peaks for SiO_2_ were observed in the XRD patterns of the composites [30,32,33,34,49]. However, the increase in the baseline within the 20–30 2θ degree region suggests the presence of amorphous silica [29,50,51]. No impurities were observed. The difference in the TEM and XRD data for the composites is explained by the fact that the SiO_2_ and ZnO oxides are really mixed on the magnetite surface and there is no clear crystal structure; some zinc oxide islands appeared as small peaks on the XRD pattern.

The XRD pattern of the modified composite exhibited the same features as the initial composite, indicating that the Fe_3_O_4_@SiO_2_@ZnO phase was maintained after activation. The average crystallite sizes of Fe_3_O_4_@SiO_2_@ZnO and Fe_3_O_4_@SiO_2_@ZnO* were determined to be 20 nm and 19 nm, respectively, using the Debye–Scherrer equation. Given that the size of the magnetite was 12.5–14.2 nm [50,51], a shell was present but was not large enough to produce silica peaks in the 21 degree 2θ region of the diffractograms [51]. These data correlate with the EDXS data and confirm the presence of iron on the surface that is not covered by shells.

### 2.4. The Textural Properties of the Synthesized Composites

**Figure 6:**
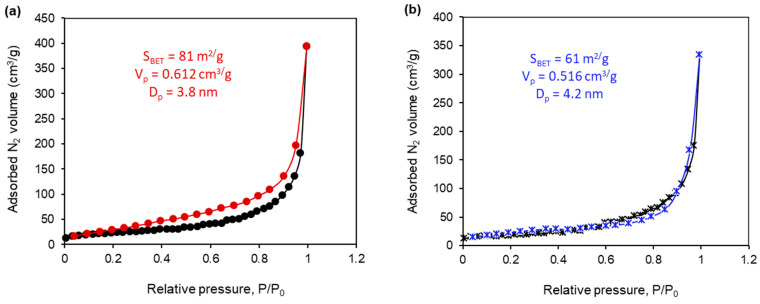
N_2_ adsorption–desorption isotherms of Fe_3_O_4_@SiO_2_@ZnO (**a**) and Fe_3_O_4_@SiO_2_@ZnO* (**b**).

N_2_ adsorption–desorption measurements were carried out to study the textural properties of the synthesized Fe_3_O_4_@SiO_2_@ZnO composite and to compare them with those of its hydrogen peroxide-modified form, Fe_3_O_4_@SiO_2_@ZnO*. The N_2_ adsorption–desorption isotherms for both the Fe_3_O_4_@SiO_2_@ZnO and Fe_3_O_4_@SiO_2_@ZnO* composites are presented in Figure 6. These isotherms displayed typical type II characteristics with an H3 hysteresis loop according to IUPAC, indicating the presence of mesopores in the samples [35,37]. The textural properties of the tested composites, including the BET surface area (S_BET_), total pore volume (V_p_), and mean pore diameter (D_p_), are listed in Figure 6. The BET surface areas of the Fe_3_O_4_@SiO_2_@ZnO and Fe_3_O_4_@SiO_2_@ZnO* composites were 81 m²/g and 61 m²/g, respectively, the pore sizes were 3.8 nm and 4.2 nm, and the total pore volumes were 0.61 cm³/g and 0.52 cm³/g, respectively. It is evident that the textural parameters for both composites were similar, with only a slight decrease in the BET surface area for the modified composite. This decrease could be attributed to the thickening of agglomerates caused by the redistribution of charges on the surface, leading to an increase in the space between pores. Consequently, the Fe_3_O_4_@SiO_2_@ZnO and Fe_3_O_4_@SiO_2_@ZnO* composites are characterized as non-porous, possessing a sorption volume formed by aggregated nanoparticles, with gaps and holes between the particles and agglomerates forming the pores.

### 2.5. Zeta Potential Measurements

**Figure 7:**
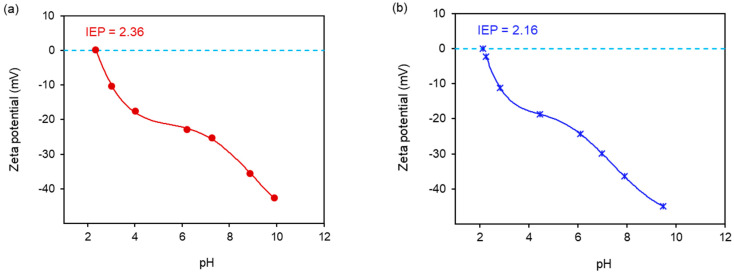
Zeta potential of the synthesized Fe_3_O_4_@SiO_2_@ZnO (**a**) and Fe_3_O_4_@SiO_2_@ZnO* (**b**) at different pH values.

The results of the zeta potential test, as a function of pH values for the two composites, are presented in Figure 7. The zeta potential values for Fe_3_O_4_@SiO_2_@ZnO and Fe_3_O_4_@SiO_2_@ZnO* were similar, falling within the ranges of −42.7 mV < ζ < 0 mV and −45.0 mV < ζ < −2.4 mV, respectively. These results indicate instability and a tendency for particles to agglomerate in both composites. An increase in pH led to a decrease in the zeta potential of Fe_3_O_4_@SiO_2_@ZnO and Fe_3_O_4_@SiO_2_@ZnO* (Figure 7), making the surfaces of the samples more negatively charged. This suggests that the particles of the composites are negatively charged due to the presence of anionic groups on their surfaces. This finding is consistent with the literature, which explains that the increase in OH^−^ ions [52,53] leads to the formation of negatively charged Si-OH [51] and/or Zn(OH)^3−^ and Zn(OH)_4_^2−^ or (≡Zn–O^−^) [54] on the composite surface. Additionally, the IR data confirmed the presence of –OH groups from oxides on the surface. The isoelectric points (IEPs) of Fe_3_O_4_@SiO_2_@ZnO and Fe_3_O_4_@SiO_2_@ZnO* were determined to be 2.36 and 2.16, respectively, indicating acidic pH values. In the case of the modified composite, the IEP was determined by interpolating the curve to zero on the pH scale from the zeta potential measurements.

### 2.6. Photoluminescence (PL) Properties

**Figure 8:**
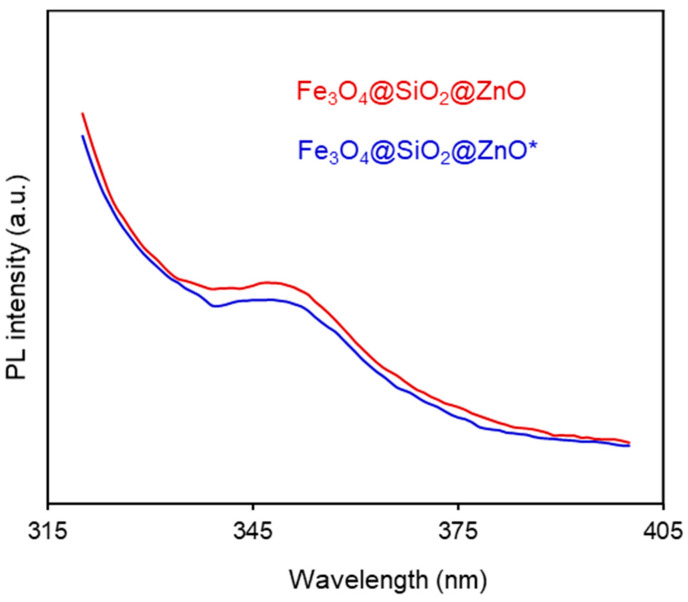
PL spectra of the Fe_3_O_4_@SiO_2_@ZnO and Fe_3_O_4_@SiO_2_@ZnO* water suspensions.

To understand the electron-hole separation efficiency in the composites, photoluminescence (PL) emission measurements were conducted on the Fe_3_O_4_@SiO_2_@ZnO and Fe_3_O_4_@SiO_2_@ZnO* composites. The resulting PL spectra are shown in Figure 8. PL emission primarily originates from the recombination of photogenerated carriers [55]. Both composites exhibited an emission peak at approximately 350 nm, whereas other researchers have reported emission peaks at 380 nm, 382 nm, 369–374 nm, and 400 nm in their respective studies [32,36,43,51]. Notably, Fe_3_O_4_@SiO_2_@ZnO* demonstrated a lower PL intensity compared to Fe_3_O_4_@SiO_2_@ZnO, indicating a higher electron-hole separation efficiency in the modified composite [56,57]. This is also indicative of prolonged carrier lifetime [32] and a lower recombination rate of electron-hole pairs [56,57]. Therefore, using H_2_O_2_ to modify the Fe_3_O_4_@SiO_2_@ZnO composite appears to be an effective alternative method for reducing the recombination process [39,58] potentially improving photocatalytic efficiency.

### 2.7. Photocatalytic Ability of the Synthesized Fe_3_O_4_@SiO_2_@ZnO and Fe_3_O_4_@SiO_2_@ZnO* Composites

**Figure 9:**
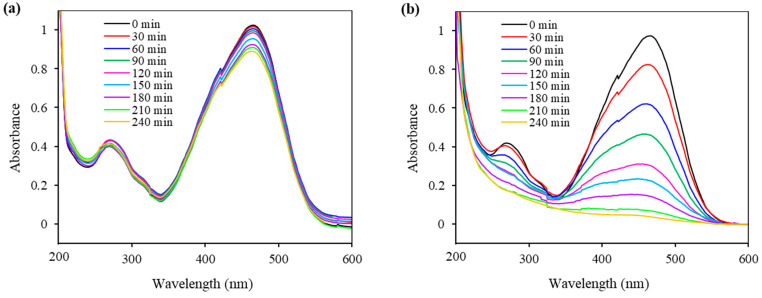
UV–vis spectra of MO during UV treatment with Fe_3_O_4_@SiO_2_@ZnO (**a**) and Fe_3_O_4_@SiO_2_@ZnO* (**b**) composites.

**Figure 10:**
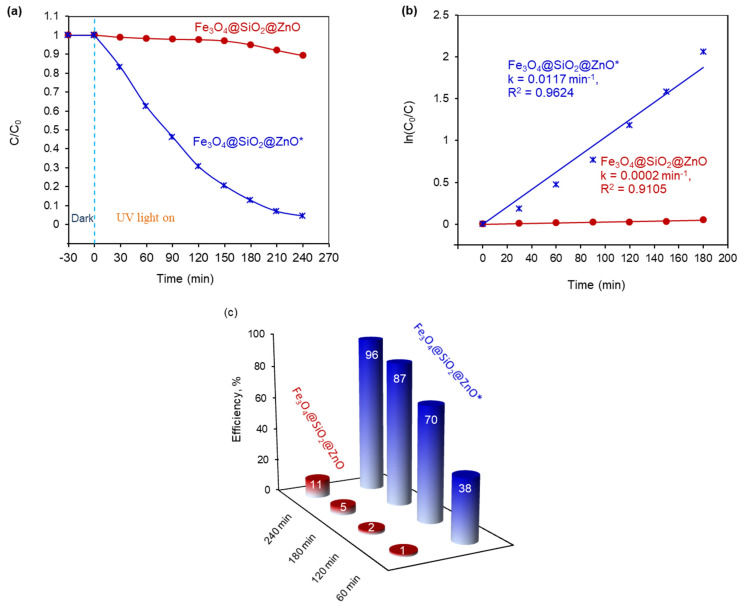
The photocatalytic degradation of MO in the presence of Fe_3_O_4_@SiO_2_@ZnO and Fe_3_O_4_@SiO_2_@ZnO* under UV treatment (**a**), with pseudo-first-order kinetic curves and corresponding reaction rates (**b**), and the percentage of photocatalytic degradation efficiency of MO (**c**).

The photocatalytic activity of the Fe_3_O_4_@SiO_2_@ZnO composite and its modified form was evaluated by the photodegradation of methyl orange (MO) under UV treatment, as shown in Figure 9 and Figure 10. MO, as previously mentioned, is a typical organic azo dye pollutant widely used in the textile industry, contributing to environmental pollution.

The changes in the absorption spectra of the MO solution over time, in the process of a photodegradation reaction using Fe_3_O_4_@SiO_2_@ZnO and Fe_3_O_4_@SiO_2_@ZnO*, are presented in Figure 9. The characteristic absorption peak of MO at 460 nm slightly decreased for Fe_3_O_4_@SiO_2_@ZnO, whereas for Fe_3_O_4_@SiO_2_@ZnO*, it continued to decrease over time and ultimately disappeared completely after 240 min. This indicates the complete removal of the azo dye using the modified composite as a photocatalyst.

The corresponding kinetic curves for MO degradation and the MO removal efficiencies over time are presented in Figure 10a,c. It is observed that, for Fe_3_O_4_@SiO_2_@ZnO, the photodegradation of MO is extremely slow, with degradation efficiencies of only 1% after 60 min, 2% after 120 min, 5% after 180 min, and 11% after 240 min. In contrast, the modification of the composite significantly enhanced azo dye removal, achieving degradation efficiencies of 38%, 70%, 87%, and at least 96% for 60, 120, 180, and 240 min, respectively. Therefore, the MO removal efficiency for Fe_3_O_4_@SiO_2_@ZnO* over 240 min was 8.7 times higher than that of Fe_3_O_4_@SiO_2_@ZnO.

The variation in the MO concentration over time during the photodegradation process using Fe_3_O_4_@SiO_2_@ZnO and Fe_3_O_4_@SiO_2_@ZnO* under UV irradiation can be aptly described by a pseudo-first-order kinetics model. This is represented by the equation ln(C/C_0_) = k_1_t, where C_0_ and C are the initial and current concentrations of MO, respectively, and k_1_ is the first-order rate constant [39]. The model yields satisfactory linear correlation coefficients (R²) of 0.9105 and 0.9624, respectively, as shown in Figure 10b. These results enabled the calculation of the pseudo-first-order rate constant k_1_, also presented in Figure 10b. The rate constant for Fe_3_O_4_@SiO_2_@ZnO* reached 0.0117 min^−1^, which was 58.5 times higher than that for Fe_3_O_4_@SiO_2_@ZnO (0.0002 min^−1^).

The low degradation removal rate of MO, an anionic dye, can be attributed to the weak Coulomb interaction between the negative surface charge of the photocatalyst and the negatively charged dye molecules, a factor that significantly affects catalytic performance [29]. However, both composites synthesized in our study, Fe_3_O_4_@SiO_2_@ZnO and Fe_3_O_4_@SiO_2_@ZnO*, exhibited similar IEPs and negative surface charges. Therefore, in our case, the recombination process plays a crucial role in determining the catalytic effectiveness. The enhanced catalytic performance observed in the H_2_O_2_-modified composite, Fe_3_O_4_@SiO_2_@ZnO*, is attributed to effective electron-hole separation and reduced recombination [59], as also confirmed by the photoluminescence study of the composites.

**Figure 11:**
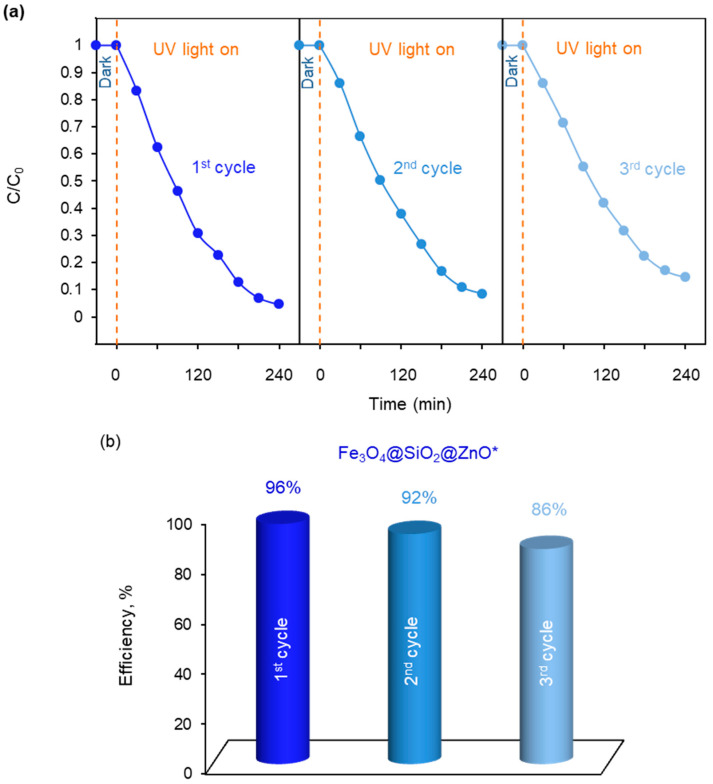
The reusability of Fe_3_O_4_@SiO_2_@ZnO* for three cycles: the curves of photocatalytic degradation of MO (**a**), and the percentage of photocatalytic degradation efficiency of MO (**b**).

The reusability of the more active photocatalyst Fe_3_O_4_@SiO_2_@ZnO*, following the photodegradation of MO, was investigated over three cycles and is presented in Figure 11. As illustrated in Figure 11b, the catalyst exhibited a decrease in performance from 96% in the first cycle to 92% and 86% in the second and third cycles, respectively. Therefore, the photocatalytic efficiency decreased slightly after each run but still achieved a value of 86% after three cycles. The obtained results indicate that Fe_3_O_4_@SiO_2_@ZnO* exhibits good cycling stability.

**Figure 12:**
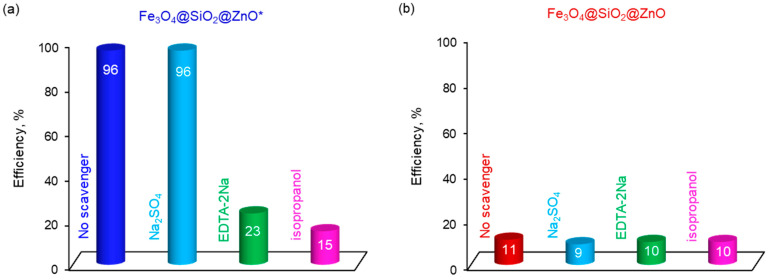
The effect of adding different scavengers on the photocatalytic degradation of MO using Fe_3_O_4_@SiO_2_@ZnO (**a**) and Fe_3_O_4_@SiO_2_@ZnO* (**b**).

To investigate the mechanism of the photocatalytic process, radical trapping experiments were conducted (Figure 12a,b). For Fe_3_O_4_@SiO_2_@ZnO*, the addition of Na_2_SO_4_ as an e^–^ scavenger did not affect the photodegradation efficiency of MO, which remained at 96%, the same as without the scavenger (Figure 12a). However, the efficiency of MO significantly decreased from 96% to 23% and 15% after the introduction of ethylenediaminetetraacetate (EDTA-2Na) and isopropanol as scavengers for h^+^ and OH˙, respectively. This suggests that OH˙ radicals can be generated from the activated form of the photocatalyst as well as from holes, with holes being the most probable dominant source of OH˙. The results indicate that the photocatalytic degradation of MO over Fe_3_O_4_@SiO_2_@ZnO* primarily involves h+ and OH˙ species, whereas e^–^ and consequently $O_{2}^{\cdot -}$ did not play a significant role in the process.

In the case of Fe_3_O_4_@SiO_2_@ZnO, the addition of e^–^, h^+^ and OH˙ scavengers did not significantly affect the process; the photodegradation efficiency decreased slightly from 11% (with no scavenger) to 9%, 10%, and 10%, respectively (Figure 12b). These results show that e^–^ and h^+^ played only a minor role in the process of radical generation, and these electron-hole pairs were most likely recombined.

### 2.8. Proposed Mechanism of the Photodegradation of MO in the Presence of Fe_3_O_4_@SiO_2_@ZnO* Composite under UV Irradiation

**Figure 13:**
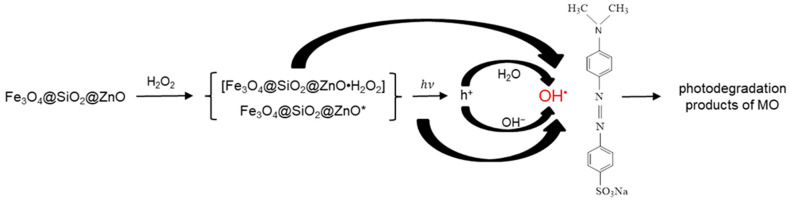
Hypothesized pathway for the UV-induced photocatalytic degradation of MO by the Fe_3_O_4_@SiO_2_@ZnO* composite.

Based on the obtained results and an analysis of the literature [9,28,31,38,39,57], a possible mechanism for the photocatalysis of MO by Fe_3_O_4_@SiO_2_@ZnO* is proposed, as illustrated in Figure 13.

The modification of the Fe_3_O_4_@SiO_2_@ZnO composite with H_2_O_2_ may occur through the adsorption of hydroperoxide molecules on the composite’s surface, forming an intermediate complex between the catalyst and hydroperoxide, [Fe_3_O_4_@SiO_2_@ZnO·H_2_O_2_] or Fe_3_O_4_@SiO_2_@ZnO*:

$$Fe_{3}O_{4}@SiO_{2}@ZnO+H_{2}O_{2}\;\to \left[Fe_{3}O_{4}@SiO_{2}@ZnO\cdot H_{2}O_{2}\right]\;or\;Fe_{3}O_{4}@SiO_{2}@ZnO^{*}.$$

In this complex, H_2_O_2_, acting as an electron acceptor, can capture electrons from the composite’s surface and generate hydroxyl radicals (OH˙) on the photocatalyst surface [38,40,60]:

$$H_{2}O_{2}+e^{-}\to OH^{-}+OH^{\cdot }.$$

Under UV irradiation, the composite can absorb light, generating electrons (e^−^) and holes (h^+^). However, in the case of the modified composite, we can assume the dominant creation of (h^+^) and only slight formation of (e^−^) as follows:

$$Fe_{3}O_{4}@SiO_{2}@ZnO^{*}+h\nu \;\to h^{+}\;\left(\mathrm{dominant}\;\mathrm{process}\right)+e^{-}\;\left(\mathrm{slight}\;\mathrm{process}\right),$$

which we can disregard. Therefore,

$$Fe_{3}O_{4}@SiO_{2}@ZnO^{*}+h\nu \;\to h^{+}.$$

In this scenario, the further generation of radicals in the photocatalytic degradation of MO in water solution can proceed through the reaction of formed holes with water molecules or hydroxyl ions (OH^−^), forming hydroxyl radicals (OH˙) as follows:

$$h^{+}+H_{2}O\to OH^{\cdot }+H^{+},$$

$$h^{+}+OH^{-}\to OH^{\cdot }.$$

If it is proposed that a negligible amount of hydrogen peroxide molecules remain on the composite surface, then under UV treatment, they should transform into OH˙ as follows:

$$H_{2}O_{2}+h\nu \to 2OH^{\cdot }.$$

The generated hydroxyl radicals (OH˙) are powerful oxidizing agents that interact with the organic pollutant MO, transforming it into photodegradation products as follows:

$$OH^{\cdot }+MO\to photodegradation\;products\;of\;MO.$$

It is important to note that the recombination of charge-carrier electron-hole pairs, releasing energy as light or heat, results in the termination of the chain reaction and reduces the efficiency of the photodegradation process as follows:

$$e^{-}+h^{+}\to heat$$

Therefore, the process of capturing and accepting electrons by H_2_O_2_ can limit this recombination process and thereby increase the photocatalytic efficiency.

In the case of an unmodified composite, when the light of energy irradiates it, Fe_3_O_4_@SiO_2_@ZnO absorbs this light, leading to the formation of charge carriers:

$$Fe_{3}O_{4}@SiO_{2}@ZnO+h\nu \;\to h^{+}+e^{-}.$$

The generated (e^−^)/(h^+^) pair may recombine, releasing energy as follows:

$$e^{-}+h^{+}\to heat.$$

Thus, according to the obtained results, it can be assumed that the high photodegradation efficiency of the modified composite, Fe_3_O_4_@SiO_2_@ZnO*, is due to the process of capturing electrons by H_2_O_2_, whereas the low efficiency of the unmodified Fe_3_O_4_@SiO_2_@ZnO may be a result of a high rate of recombination or a very slow rate of radical formation, limiting the photodegradation process.

Table 1 summarizes the results of the investigations into the photocatalytic degradation of methyl orange using magnetic catalysts containing the magnetite Fe_3_O_4_ and semiconductor ZnO, as well as other components presented in previous works [28,29,30,37,61], and compares these with data obtained in the current study. It is observed that Fe_3_O_4_@SiO_2_@ZnO and Fe_3_O_4_@SiO_2_@ZnO@La, as reported in [30], exhibited efficiencies of 88% and 94%, respectively, for 100 min at a dye concentration 4.5 times lower and a catalyst content 16 times higher than those used in this work. The rate constant for photocatalysis by Fe_3_O_4_@SiO_2_@ZnO in [29] reached 0.004 min^−1^ at an MO concentration of 5 mg/L, with the same composite concentration, which is 2.9 times lower than in our case. At a nearly identical dye concentration, but with 1.25 times less photocatalyst content, Fe_3_O_4_@ZnO/PW achieved a rate constant of 0.0138 min^−1^ [37], nearly equal to that of Fe_3_O_4_@SiO_2_@ZnO*. Fe_3_O_4_/ZnO-GO showed a dye removal efficiency of 92.8% and a rate constant of 0.05558 min^−1^ with a significantly lower photocatalyst amount (five times less), but also a lower dye concentration (4.13 times less). Therefore, it is evident that the synthesized modified composite Fe_3_O_4_@SiO_2_@ZnO* generally demonstrated superior photocatalytic performance in removing MO, particularly at a higher azo dye concentration (specifically 13.5 mg/L); in other studies the dye concentration was lower, ranging from 3 mg/L to 13.08 mg/L.

## 3. Materials and Methods

### 3.1. Chemicals

The chemical reagents used in this study were sourced from various suppliers. Methyl orange (MO, C_14_H_14_N_3_NaO_3_S), a dye, was obtained from Lachema in the Brno, Czech Republic. Iron(II) chloride tetrahydrate (FeCl_2_·4H_2_O, 99%), iron(III) chloride hexahydrate (FeCl_3_·6H_2_O, 99%), potassium sulfate (Na_2_SO_4_, 99%), zinc acetate dihydrate (Zn(CH_3_COO)_2_·2H_2_O, 99%), and disodium ethylenediaminetetraacetate (EDTA-2Na, C_10_H_14_N_2_Na_2_O_8_·2H_2_O, 99%) were supplied by CentralChem, Bratislava, Slovakia. Tetraethyl orthosilicate (TEOS, Si(OC_2_H_5_)_4_, 98%) came from Sigma-Aldrich. Ethanol (C_2_H_5_OH, EtOH, 96%), isopropanol ((CH_3_)_2_CHOH, 99.5%), and a 25% solution of ammonium hydroxide (NH_4_OH) were acquired from microCHEM, Pezinok, Slovakia, and Lach-Ner, Neratovice, Czech Republic, respectively. Deionized water (DI-H_2_O) was utilized for preparing all aqueous solutions.

### 3.2. Synthesis of Magnetite Fe_3_O_4_ Nanoparticles

A total of 500 mL of deionized water was heated to 80 °C. Then, 14.17 g of FeCl_3_·6H_2_O was added to this hot water, under vigorous stirring for 5 min. After that, 5.49 g of FeCl_2_·4H_2_O was added to the solution, continuing the stirring and heating for an additional 5 min. Next, 50 mL of a 25% NH_4_OH solution was added dropwise, and the mixture was stirred for 30 min. Once the solution cooled to room temperature, the magnetic particles were collected using a magnet, followed by washing ten times with DI-H_2_O and twice with EtOH. The magnetic particles were then covered with 250 mL of EtOH and stored in the refrigerator. The concentration of magnetite in the solution was determined by the weight method and found to be 25.78 mg/mL.

### 3.3. Synthesis of Fe_3_O_4_@SiO_2_@ZnO Composite

Initially, 10 mL of a magnetite particle solution of Fe_3_O_4_ (25.78 mg/mL) was dispersed in 100 mL of EtOH. Subsequently, 3.86 g of Zn(CH_3_COO)_2_·2H_2_O was dissolved in 50 mL of EtOH. Then, 2.8 mL of TEOS and the prepared zinc acetate solution were added to the Fe_3_O_4_ solution under continuous mechanical stirring and heated to 60 ºC. Afterward, 30 mL of 25% NH_4_OH was gradually added to the final solution over a period of 4 h while stirring. The Fe_3_O_4_@SiO_2_@ZnO sample was then separated using a magnet, washed with DI-H_2_O and EtOH, dried at 100 °C for 12 h, and finally calcined at 300 °C for 30 min.

**Figure 14:**
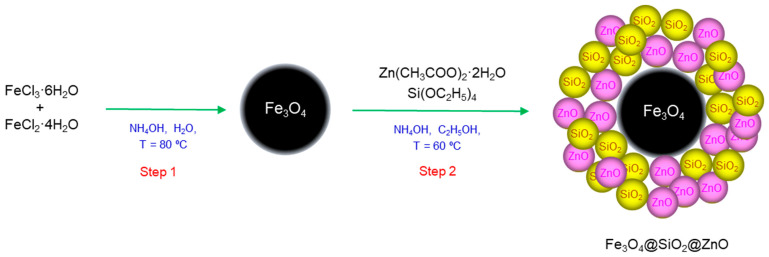
Schematic illustration of the two-step synthesis of the Fe_3_O_4_@SiO_2_@ZnO composite.

The synthesis procedure is schematically illustrated in Figure 14.

### 3.4. Synthesis of Fe_3_O_4_@SiO_2_@ZnO* Sample

A total of 0.05 g of Fe_3_O_4_@SiO_2_@ZnO was added to 50 mL of a 0.069 M H_2_O_2_ solution. The suspension was subjected to ultrasound treatment for 5 min, followed by UV irradiation with stirring for 15 min. After that, the suspension was collected by centrifugation and dried at room temperature for 12 h.

### 3.5. Methods

The morphology of the synthesized samples was characterized using a field emission scanning electron microscope (MIRA 3 FE-SEM, TESCAN, Brno, Czech Republic), equipped with a high-resolution cathode (Schottky field emitter) and a three-lens Wide Field Optics™ design, and transmission electron microscope (JEOL 2100F UHR), operated at 200 kV with a Field Emission Gun. The Fourier transform infrared spectrum of the sample was obtained using a Bruker TENSOR 27 FT-IR spectrometer and the KBr pellet technique. XRD measurements were conducted in reflection Bragg–Brentano geometry using a BRUKER D2-Phaser diffractometer (Germany). Diffraction experiments utilized Cu-Kα radiation (λ = 0.154 nm), covering a 2θ range of 20–70 degrees with a step size of 0.02 degrees. These were recorded using a fast microstrip detector. The BET surface areas of the samples were determined by the N_2_ adsorption–desorption method using a NOVA 1200e Surface Area and Pore Size Analyzer from Quantachrome Instruments, Beach, FL, USA. Zeta potentials were measured using a Zetasizer Nano ZS (Malvern, UK) in a 1 g/L sample suspension in a 0.001 M NaNO_3_ solution. The photoluminescence measurements were performed at room temperature using a photon counting spectrofluorometer PC1 (ISS) at an excitation wavelength of 300 nm, with a 300 W xenon lamp used as the excitation source.

### 3.6. Photocatalytic Degradation Activities of Fe_3_O_4_@SiO_2_@ZnO Composite and Its Activated Form

The photocatalytic performance of the synthesized Fe_3_O_4_@SiO_2_@ZnO and Fe_3_O_4_@SiO_2_@ZnO* was evaluated using MO as a model dye pollutant for photodegradation under UV light irradiation (9 W, λ = 369 nm, UVA-Radiator 368, NBB Bohemia s.r.o., Presov, Slovakia). Typically, 50 mg of the photocatalyst was dispersed in 50 mL of an aqueous MO solution with a concentration of 13.5 mg/L. To achieve adsorption–desorption equilibrium between the dye and the photocatalyst, their suspension was magnetically stirred in dark conditions for 15 min before the UV light was turned on. The photodecomposition of methyl orange under the conditions of our experiment without catalysts was 4% within 240 min, as well as 10% when using Fe_3_O_4_. The photodegradation of MO using Fe_3_O_4_@SiO_2_@ZnO and Fe_3_O_4_@SiO_2_@ZnO* was also investigated by adding scavengers (2 mM), specifically, Na_2_SO_4_ as an electron scavenger, EDTA-2Na as a hole scavenger, and isopropanol as a hydroxyl radical scavenger. Additionally, the stability of the photocatalyst Fe_3_O_4_@SiO_2_@ZnO* was evaluated over three cycles. At specific intervals during photoirradiation, 2.5 mL of the suspension was collected and filtered through a 0.22 µm filter (AZ Chrom™, Bratislava, Slovakia). The concentration of the remaining MO was determined by measuring the absorbance of the filtered solutions (λ_max_ = 460 nm) using a UV–vis spectrophotometer from Helios Gamma (ThermoElectron Corporation, Rugby, UK).

## 4. Conclusions

A two-step facile method for synthesizing the Fe_3_O_4_@SiO_2_@ZnO composite was successfully implemented. Additionally, a straightforward method for its modification with H_2_O_2_, creating the highly active novel photocatalyst Fe_3_O_4_@SiO_2_@ZnO*, was developed. The modified composite, Fe_3_O_4_@SiO_2_@ZnO*, exhibited enhanced photocatalytic activity, achieving a 96% removal of methyl orange in 240 min during photodegradation under UV irradiation, compared to only 11% for Fe_3_O_4_@SiO_2_@ZnO. The first-order kinetic constant for photodegradation by Fe_3_O_4_@SiO_2_@ZnO* was found to be almost 58.5 times higher than that of Fe_3_O_4_@SiO_2_@ZnO. Fe_3_O_4_@SiO_2_@ZnO* maintained a photodegradation efficiency of over 86% after three cycles of dye removal. This method of modification by using H_2_O_2_ proves to be an effective way to improve photocatalyst performance by enhancing the separation of electron-hole pairs and reducing their recombination, as evidenced by photoluminescence results. The primary function of H_2_O_2_ in the modification process is to capture and accept electrons from the composite’s surface, thereby generating hydroxyl radicals (OH·) that break down the dye. Free radical trapping experiments indicated that h^+^ and OH· were the main active species affecting the efficiency of MO degradation using Fe_3_O_4_@SiO_2_@ZnO*. Hence, the findings of this study show that modifying Fe_3_O_4_@SiO_2_@ZnO with H_2_O_2_ significantly enhances its performance, suggesting that the modified composite, Fe_3_O_4_@SiO_2_@ZnO*, holds potential for use in the degradation of azo dyes.

## Figures and Tables

**Table 1:** Performance of different photocatalysts containing magnetite and zinc oxide in MO degradation.

Photocatalyst	Photocatalyst Dosage, mg	MO Concentration	MO Volume, mL	Light Source	Time, min	Efficiency, %	Rate Constant, k_1_, min^−1^	Ref.
Fe_3_O_4_/ZnO-GO	20	1 × 10^−5^ M	100	300 W, Xe lamp	150	92.8	0.05558	[57]
Fe_3_O_4_@ZnO/PW	40	4 × 10^−5^ mol/L	50	2 × 20 W, white LED lamps	180	92.3	0.0138	[35]
Fe_3_O_4_@SiO_2_@ZnO	100	5 mg/L	100	100 mW cm^−2^, mercury lamp	60	–	0.004	[27]
Fe_3_O_4_@SiO_2_@ZnO@La	800	3 mg/L	50	300 W, mercury lamp	100	94	–	[28]
Fe_3_O_4_@SiO_2_@ZnO	800	3 mg/L	50	300 W, mercury lamp	100	88	–	[28]
Fe_3_O_4_@SiO_2_@ZnO*	50	13.5 mg/L	50	9 W, UV	240	96	0.0117	This work

## Data Availability

The data are contained within the article.
